# The effect of physical activity on homework effort of middle school students: the chain mediating effect of executive function and positive homework emotions

**DOI:** 10.3389/fpsyg.2026.1749045

**Published:** 2026-02-06

**Authors:** Yu Su, Renren Zhang, Pandeng Chen, Yaling Zhou, Yu Zhou, Xiangyu Wang

**Affiliations:** 1 School of Physical Education, Sichuan Normal University, Chengdu, China; 2 Sichuan Normal University Sports Education Research Center, Chengdu, China; 3 School of Foreign Languages, Sichuan University Jinjiang College, Meishan, China; 4 School of Physical Education, China University of Mining and Technology, Xuzhou, China

**Keywords:** executive function, homework effort, middle school students, physical activity, positive homework emotions

## Abstract

**Objective:**

Homework effort is widely recognized as a key factor influencing academic performance. This study explores the profound association between physical activity (PA) and homework effort among middle school students, along with the mediating roles of executive function and positive homework emotions between PA and homework effort.

**Methods:**

A total of 1,776 Chinese students in Grades 7–9 of China’s compulsory education system were surveyed using the Physical Activity Questionnaire, Homework Effort Scale, Executive Function Scale, and Positive Homework Emotion Scale. A chain mediation model and the Bootstrap method were employed to test the mediating effects.

**Results:**

Physical activity, executive function, positive homework emotions, and homework effort were all significantly positively correlated (*p* < 0.001). PA emerged as a potential direct predictor of homework effort (*β* = 0.09, *p* < 0.001). Executive function and positive homework emotions exerted significant mediating effects on the relationship between physical activity and homework effort (*p* < 0.001). These mediating effects were manifested through three pathways: the mediating role of executive function (CI = 0.08 to 0.21); the mediating role of positive homework emotions (CI = 0.22 to 0.37); the chain mediating role of executive function and positive homework emotions (CI = 0.03 to 0.07).

**Conclusion:**

The chain mediation model of executive function and positive homework emotions between PA and homework effort reveals a novel mechanism underlying the association between PA and homework effort in middle school students. These findings suggest that middle school students should be encouraged to engage in physical activity to enhance their executive function and positive homework emotions, thereby exerting a positive effect on homework effort.

## 1 Introduction

Teachers, parents, and students across most countries regard homework effort as a crucial factor influencing academic achievement (Trautwein et al., 2006). Recent findings reveal that adolescents aged 11–15 years struggle with learning difficulties and anxiety associated with schoolwork pressure (World Health Organization Regional Office for Europe and Health Behavior in School-Aged Children [HBSC], 2024), coupled with the prevalence of “pseudo-engagement” and “pretended effort” in learning and homework completion (Sang, 2019; Li, 2022). Meanwhile, insufficient physical activity among adolescents has become a global issue, with over 80% of adolescents aged 11–17 worldwide failing to meet the physical activity criteria set by the World Health Organization (Cardon and Salmon, 2020). It is evident that physical activity, learning engagement, and homework effort in secondary school have emerged as key concerns for societies around the world.

Physical activity (PA) refers to any bodily movement that requires energy expenditure (Wang et al., 2019). Surveys in China show that overall participation in PA among children and adolescents remains relatively low (Guo, 2016). Furthermore, studies consistently demonstrate that adolescents’ engagement in PA at varying intensities exerts a positive effect on physical health (Gao et al., 2024), and contributes to the development of the overall physical fitness (Yang et al., 2024). Beyond physical health benefits, a growing body of research has pointed out that PA, including participation in sports, can enhance academic performance in school-aged children (Wang et al., 2021; Owen et al., 2021; Francesca and Francesco, 2023). After-class homework, as an extension of classroom instruction, is defined as “tasks assigned by school teachers to be completed after school or during non-school hours” (Cooper, 2001). Homework effort is recognized as a significant predictor of students’ academic performance (José et al., 2015; Ozyildirim, 2022; Zhang et al., 2025). It refers to the investment and concentration that students demonstrate during homework completion (Trautwein et al., 2006). Due to China’s exam-oriented education system, homework takes up a considerable portion of students’ after-school time. Currently, homework-related behaviors among middle school students, such as insufficient effort, poor concentration, and anxiety, have raised widespread concerns among families, schools, and society (Liu et al., 2016; Jia et al., 2023). Thus, there is a theoretical basis for the potential association between PA and homework effort. Logically, PA can boost students’ initiative and concentration during homework by regulating their emotional states and alleviating academic pressure, thereby exerting a positive effect on homework effort. To date, research on homework effort has primarily focused on the effect of individual factors (e.g., conscientiousness and motivation), environmental factors (e.g., teacher support and homework quality), and family-related factors on homework effort (Liu et al., 2016). The effect of physical activity on homework effort remains underexplored.

Executive function (EF), a higher-order cognitive ability, has recently attracted substantial research attention in cognitive and exercise psychology. It comprises a set of goal-oriented behavioral processes regulated by the prefrontal cortex, including three core components: inhibitory control, working memory and cognitive flexibility (Julia et al., 2016). Studies demonstrate that EF exerts positive effects on adolescents’ attention, learning behaviors, academic emotions, revealing a theoretical link between EF and homework effort (Yang, 2021; Zhu and Zhao, 2023; Xing et al., 2023). As a distinct type of academic emotion, homework emotions deserve particular attention. Homework emotions are emotions associated with homework, including both positive and negative dimensions (Thomas et al., 2012). Empirical evidence indicates that homework emotions are a critical factor influencing students’ homework effort, with positive homework emotions exerting a positive effect on homework effort (Liu et al., 2013, 2016). Xing et al. (2023) identified a positive correlation between EF and positive homework emotions. Furthermore, research demonstrates that adolescents’ participation in physical activity can enhance EF (Wang et al., 2019) and improve emotional states (Zhang et al., 2020). Collectively, these findings suggest that PA can effectively enhance adolescents’ EF and emotional states. Theoretical links exist between EF and both homework emotions and homework effort; additionally, homework emotions serve as a positive predictor of homework effort. Thus, there is a potential theoretical correlation among four core constructs: PA, EF, positive homework emotions, and homework effort. Logically, it is plausible that PA exerts an indirect positive effect on homework effort via the mediating roles of EF and positive homework emotions. Despite these findings, research gaps remain. First, the relationship between EF and homework effort is unexplored. Second, little is known about whether EF and positive homework emotions exert a synergistic effect on homework effort. Third, it is unclear whether physical activity affects homework effort through the mediating roles of EF and positive homework emotions.

## 2 Theoretical basis and research hypothesis

### 2.1 The relationship between physical activity and homework effort

Homework including both in-class and after-class assignments is an important pedagogical tool to help students consolidate knowledge learned in class. It is found that middle school students frequently demonstrate insufficient effort, inattentiveness, and anxiety during homework completion, which has raised growing concerns among families, schools, and society at large. Regarding factors influencing homework effort, prior research has identified three primary categories: individual, environmental and family-related factors. In terms of individual factors, physical activity (including sports participation) has received limited empirical attention as a potential predictor of homework effort. Previous research indicates a significant positive correlation between homework effort and academic performance, where greater effort is associated with better academic performance (Ozyildirim, 2022; Zhang et al., 2025). Wunsch et al. (2021) and Wang et al. (2021) point out that physical activity is positively correlated with homework performance and enhances overall academic performance. Given that homework effort is a key factor positively correlated with academic performance, and physical activity also shows a positive correlation with academic performance, this study hypothesizes that physical activity may positively affect homework effort. Therefore, Hypothesis 1 is proposed: Physical activity positively predicts homework effort (H1).

### 2.2 The mediating effect of executive function

The working memory model and Baddeley’s central executive hypothesis posit that EF is a prerequisite for effective learning (Case, 1985; Liu et al., 2013). Empirical evidence indicates that adolescent engagement in physical activity, regardless of the duration, intensity, or type, can enhance EF to varying degrees (Wang et al., 2019). Furthermore, research demonstrates that EF not only exerts a direct effect on academic performance (Ariel et al., 2018), but also mediates the relationship between PA and academic performance (Schmidt et al., 2017; Wen et al., 2018). Moreover, cognitive flexibility – a core component of EF – is critical for sustaining concentration (Schmidt et al., 2017), and physical exercise enhances both concentration and cognitive flexibility in adolescents (Wu and Fu, 2024). These findings suggest a theoretical link between EF and homework effort. Given that PA enhances EF, concentration, and academic performance, we speculate that EF mediates the relationship between PA and homework effort. Based on this, hypothesis 2 is proposed: EF mediates the relationship between PA and homework effort (H2).

### 2.3 The mediating effect of positive homework emotions

Emotions experienced by students during homework, such as joy and anger, are basic emotions in psychology (Thomas et al., 2012). Research confirms a significant positive correlation between positive homework emotions and homework effort in middle school students (Dettmers et al., 2011; Liu et al., 2016). Physical activity is widely recognized as an effective strategy for promoting physical and mental health. Specifically, physical activity can improve overall emotional states (Zhang et al., 2020) and enhance emotional regulation abilities (Aslinejad et al., 2024). These findings suggest that physical activity can play a positive role in improving and regulating emotions. Given that positive homework emotions are predictive variables to homework effort, it is reasonable to hypothesize that they mediate the relationship between physical activity and homework effort. Thus, hypothesis 3 is proposed: Positive homework emotions mediate the relationship between physical activity and homework effort (H3).

### 2.4 The chain mediating effect between executive function and positive homework emotions

**Figure 1:**
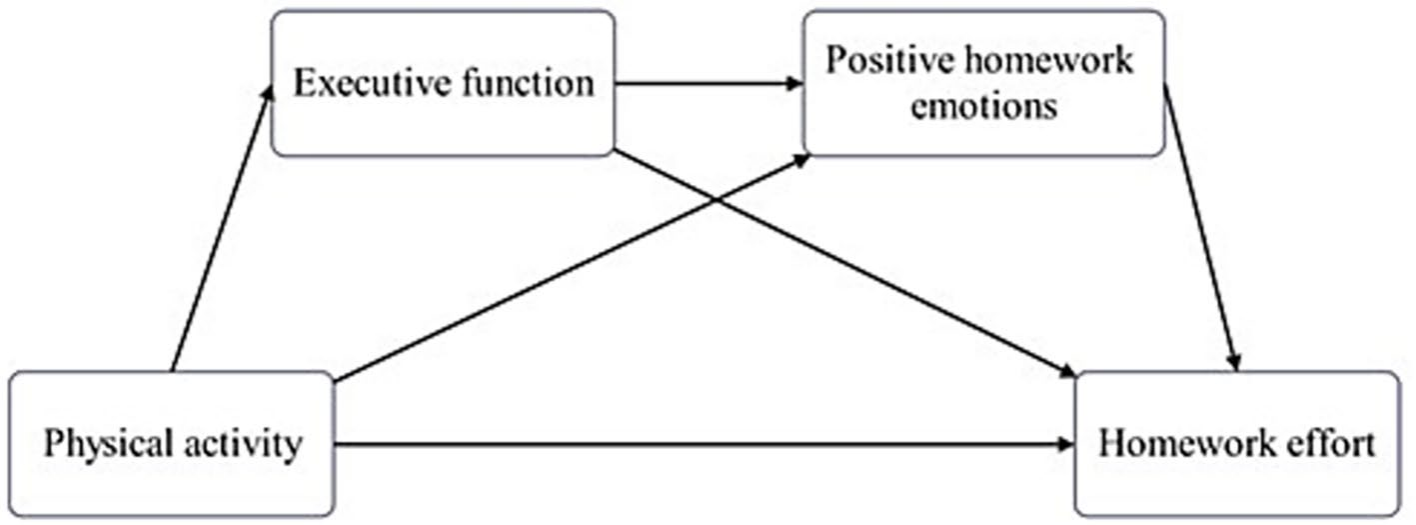
Hypothetical diagram of the chain mediation model.

According to the Control-Value Theory of Achievement Emotions, students’ cognitive abilities mediate the affect of environmental factors on academic emotions (Reinhard, 2006). Studies show that EF significantly predicts emotions (Ma et al., 2024) and mediates the effect of family-related factors on academic emotions (Xing et al., 2023), indicating its indispensable role in regulating emotional responses. Based on this analysis, an indirect pathway connects middle school students’ physical activity to homework effort: PA affects both EF and emotional states; EF correlates with emotions; and positive homework emotions positively predict homework effort. Thus, hypothesis 4 is proposed: EF and positive homework emotions play a chain mediating role between PA and homework effort (H4). Chain mediation refers to a sequential mediation process in which multiple mediating variables form a causal pathway, and the predictor variable influences the outcome variable indirectly through this chain (Ye et al., 2016). Based on these hypotheses, a conceptual model is constructed (Figure 1).

## 3 Research methods

### 3.1 Research participants

Participants in this study were Chinese students at Level Four of compulsory education. According to the Curriculum Standards for Physical Education and Health in Compulsory Education (2022 Edition) issued by the Ministry of Education in China, Level Four corresponds to Grades 7 to 9, the middle school years (Ji, 2022). A total of 2,043 questionnaires were distributed, and 1,776 valid ones were recovered, yielding an effective response rate of 86.93%. Participants’ age ranged from 12 to 14 years. Of the valid respondents, 867 were female, accounting for 48.82% of the total valid sample. This study obtained ethical approval from the Ethics Committee of Sichuan Normal University (2025LSTY001). All participants participated voluntarily, and their parents provided written informed consent.

The inclusion criteria for the sample were students: (a) possessing good proficiency in Chinese listening, speaking, reading, and writing; (b) obtaining signed informed consent from parents/legal guardians. Exclusion criteria were students in special education (e.g., physical disabilities, intellectual disabilities, and developmental defects).

### 3.2 Research tools

#### 3.2.1 Physical activity questionnaire

The Physical Activity Questionnaire (PAQ), originally developed by Kowalski et al. (2004) and later revised by Guo (2016), was used to assess middle school students’ participation in physical activity. This PAQ was specifically designed for children and adolescents aged 7 to18 years. It comprised 9 items, and each item was rated on a 5-point Likert Scale. Participants were instructed to recall their engagement in physical activity over the past 7 days when completing the PAQ. Based on their total scores, participants were categorized into three physical activity levels: high (PAQ > 3), moderate (2 < PAQ ≤ 3), and low (PAQ ≤ 2). In this study, Cronbach’s *α* coefficient of the questionnaire was 0.86. The confirmatory factor analysis fitting index was: *χ*^2^/df = 3.31, GFI = 0.99, TLI = 0.99, RMSEA = 0.04, RMR = 0.03, indicating that the questionnaire had good reliability and validity.

#### 3.2.2 Homework effort scale

The Homework Effort Scale for Junior Middle School Students, originally complied by Trautwein et al. (2006) and revised by He et al. (2021), was used to measure middle school students’ homework effort. The scale consists of 7 items across two dimensions: investment and concentration. A 4-point Likert score (1 = Completely Not Conforming; 4 = Completely Conforming) was adopted. Higher total scores are indicative of greater student effort devoted to homework. In this study, Cronbach’s *α* coefficient of the scale was 0.77. The fitting index of confirmatory factor analysis of the scale was: *χ*^2^/df = 1.348, GFI = 0.99, TLI = 0.99, RMSEA = 0.01, RMR = 0.01, indicating that the scale has good reliability and validity.

#### 3.2.3 Executive function scale

The Executive Function Scale developed by Huang et al. (2014) was employed to measure the executive function of Chinese middle school students. It includes 21 items across three dimensions: working memory, cognitive flexibility, and inhibitory control, with 7 items per dimension. This scale uses a 3-point Likert score (1 = never; 2 = sometimes; 3 = often). The scale is reverse-scored so that a higher score reflects a poorer performance of the participants’ executive functions, whereas a lower score indicates a better performance of their executive functions. In this study, Cronbach’s α coefficient of the scale was 0.95. The fitting index of confirmatory factor analysis of the scale was: *χ*^2^/df = 4.86, GFI = 0.96, TLI = 0.97, RMSEA = 0.05, RMR = 0.01, indicating good reliability and validity.

#### 3.2.4 Positive homework emotions subscale

This study used the positive homework emotions subscale from the Homework Emotions Scale developed by Thomas et al. (2012) to measure middle school students’ positive homework emotions. This scale is applicable for evaluating homework emotions in subjects such as mathematics, physics, chemistry, and languages. The subscale comprises two dimensions – Pleasure and Pride – each consisting of 4 items. It uses a 5-point Likert scale from 1 (strongly disagree) to 5 (strongly agree), where higher scores indicate stronger positive homework emotions. In this study, Cronbach’s *α*coefficient of the scale was 0.98. The confirmatory factor analysis fitting index was: *χ*^2^/df = 3.72, GFI = 0.99, TLI = 0.99, RMSEA = 0.04, RMR = 0.01, indicating that the scale has good reliability and validity.

### 3.3 Procedure

The research team first obtained approval and support from the administration departments of the sampled schools. Subsequently, informed consent forms were distributed to all students meeting the sample inclusion criteria and eligible for the survey. Students could only proceed to complete the questionnaire after their legal guardians had signed the consent forms.

Data collection was conducted from March to April 2025. Questionnaires were administered during students’ Information Technology classes (per their timetables), with the principal researcher overseeing the entire survey process.

Questionnaires were distributed via the Wenjuanxing platform. Prior to each survey, the principal researcher arrived early at the school’s computer lab and, with the Information Technology teacher’s assistance, verified that all equipment and network connections were functioning properly. Completing all questionnaire sections took approximately 25 min, including 5 min for survey instructions and a summary.

### 3.4 Statistical analysis

This study has adopted the software SPSS 26.0 and AMOS 24.0 for all data analyses. After data collection, all the data were processed as follows: (1) reliability and validity of the tools were assessed using Cronbach’s α and confirmatory factor analysis. (2) Common method bias was examined using Harman’s single-factor test. (3) Descriptive statistics were used to summarize the variables, with continuous variables presented as mean (M) ± standard deviation (SD). (4) Pearson correlation analysis was conducted to examine relationships among physical activity, executive function, positive homework emotions, and homework effort. (5) Univariate linear regression analyzed the influence of independent variables on the dependent variable, with results reported as standardized coefficients (*β*) and coefficients of determination (*R*^2^). (6) The mediating effects of executive function and positive homework emotions were tested using the PROCESS macro (version 4.0) and the Bootstrap method. A mediating effect was considered significant if its 95% confidence interval did not include zero (Hayes, 2013). The statistical significance level was set at *p* < 0.05.

## 4 Research results

### 4.1 Common method bias test

**Figure 2:**
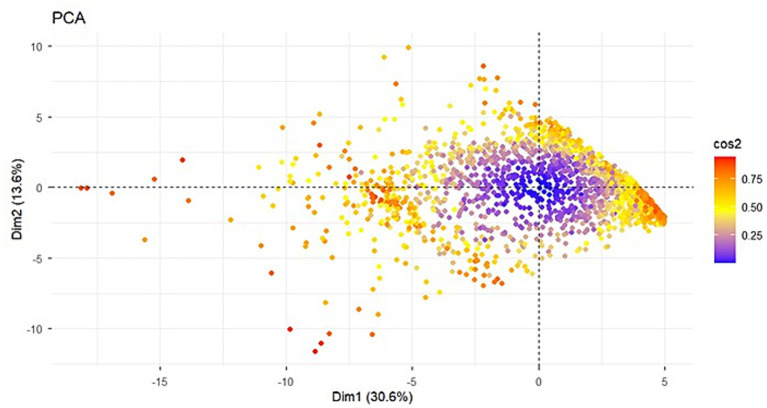
Plot of principal component analysis.

Common method bias was assessed using Harman’s single-factor test. Specifically, an unrotated principal component analysis was conducted on all 45 items from the four scales. Factors with eigenvalues greater than 1 were extracted, and the variance explained by the first factor was examined. The variance explained by the first factor was 30.6% (Figure 2), lower than the critical threshold of 40%. Thus, no serious common method bias exists (Zhou and Long, 2004).

### 4.2 Descriptive statistics and correlation analysis of physical activity, executive function, positive homework emotions, and homework effort

**Figure 3:**
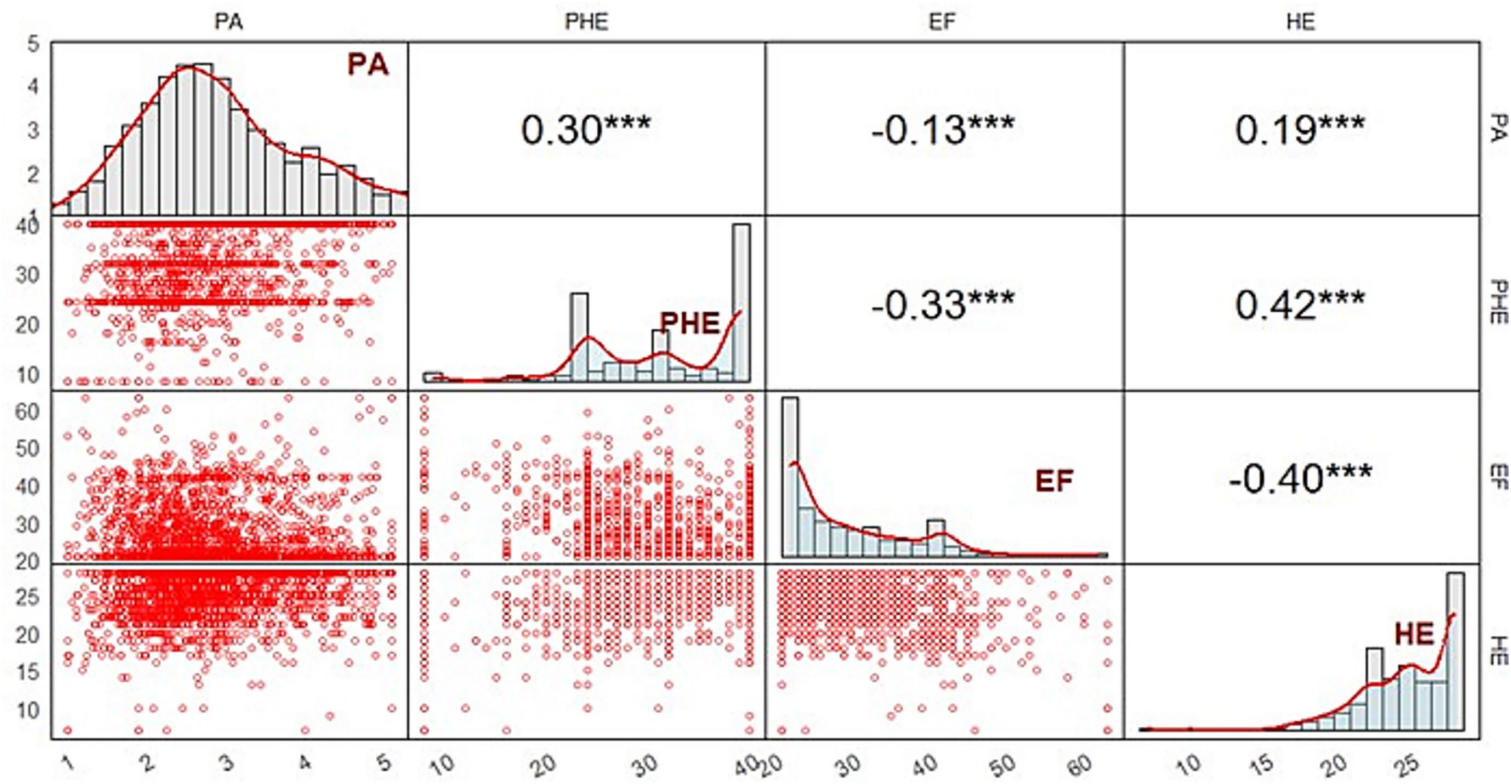
Data visualization of correlation coefficient and associated measurement sample distributions. ***p < 0.001.

Descriptive statistics of PA, EF, positive homework emotions and homework effort are as follows: the average total score of PA was 2.88 ± 0.89, the average total score of EF was 28.44 ± 8.52, the average total score of positive homework emotions was 31.76 ± 8.00, and the average total score of homework effort was 24.73 ± 3.33. The scores indicated that PA was at a moderate level (2 < PAQ ≤ 3). Pearson correlation results (Figure 3) revealed that PA and positive homework emotions were significantly positively correlated with homework effort, EF was significantly negatively correlated with PA, homework effort, and positive homework emotions, and there is a significant positive correlation between PA and positive homework emotions.

### 4.3 Regression analysis of physical activity, executive function, and positive homework emotions on homework effort

Three separate regression analyses were conducted with homework effort as the dependent variable, and PA, EF, and positive homework emotions as independent variables, respectively. The results indicated (Table 1) that PA, EF, and positive homework emotions are each potential predictors of homework effort (*p* < 0.001). The regression models explained 4, 16, and 18% of the variance in homework effort, respectively.

**Table 1:** Separate regression analysis of physical activity, executive function, and positive homework emotions on homework effort.

Variable	Homework effort					
	*B*	SE	*β*	*T*	*F*	*R* ^2^
Constant	22.65	0.26				
Physical activity	0.72	0.09	0.19	8.27	68.38	0.04***
Constant	29.17	0.25				
Executive function	−0.16	0.01	−0.40	−18.34	336.25	0.16***
Constant	19.13	0.29				
Positive homework emotions	0.18	0.01	0.42	19.71	388.64	0.18***

*R*^2^ = variance explained. *β* = standardized coefficient. ***p < 0.001.

### 4.4 Examination of the mediating effect of executive function and positive homework emotions

With participants’ gender, grade included as control variables, PA as the independent variables, EF and positive homework emotions as mediating variables, and homework effort as the dependent variable, a chain mediation effect was tested using Model 6 of the SPSS macro-PROCESS 4.0 (see Table 2 for details). The results show that PA can significantly and positively predict EF (*β* = −0.13, *p* < 0.001). After incorporating PA and EF into the regression equation to predict positive homework emotions, each showed a significant positive predictive effect (*β* = 0.25, *p* < 0.001; *β* = −0.28, *p* < 0.001). After integrating three variables—PA, EF, and positive homework emotions—into the regression equation, each exerted a significant positive influence (*β* = 0.09, *p* < 0.001; *β* = −0.29, *p* < 0.001; *β* = 0.31, *p* < 0.001). These results clarified the relationships among the variables. Among these variables, PA had a significant positive predictive effect on homework effort. Therefore, hypothesis 1 (H1) was verified.

**Table 2:** Regression analysis of variable relationships in the mediation model (*N* = 1776).

Regression equation		Overall fit coefficient			Regression coefficient significance		
Outcome variable	Predictor variable	*R*	*R* ^2^	*F*	*β*	SE	*t*
EF	PA	0.13	0.02	10.84***	−0.13	0.24	−5.39***
PHE	PA	0.43	0.19	102.43***	0.25	0.20	10.94***
	EF				−0.28	0.02	−13.79***
HE	PA	0.52	0.27	129.42***	0.09	0.08	4.08***
	EF				−0.29	0.01	−13.24***
	PHE				0.31	0.01	13.86***

PA = physical activity. EF = executive function. PHE = positive homework emotions. HE = homework effort. *R*^2^ = variance explained. β = standardized coefficient. ***p < 0.001.

The mediating effects were tested using Bootstrap method. As shown in Table 3, the total effect of PA on homework effort was 0.82. The 95% confidence interval did not contain zero, indicating a significant total effect of PA on homework effort and a significant mediating effect of the two variables on homework effort. The direct effect of PA on homework effort was 0.34. The Bootstrap 95% confidence interval did not include zero, showing a significant direct effect of PA on homework effort, and the effect size was 41.46%. The mediating effect value of EF and positive homework emotions was 0.48. The Bootstrap 95% confidence interval did not include zero, and the effect size was 58.54%. The results revealed that PA had a significant direct effect on homework effort, and EF and positive homework emotions partially mediate the relationship between PA and homework effort. Three mediating paths were identified (Bootstrap 95% confidence interval excluded zero for all): Path 1: PA → EF → Homework effort. The mediating effect of EF was significant (the mediating effect value was 0.14, accounting for 17.07% of the total mediating effect). Path 2: PA → Positive homework emotions → Homework effort. The mediating effect of positive homework emotions was significant (the mediating effect value was 0.29, accounting for 35.37% of the total mediating effect). Path 3: PA → EF → Positive homework emotions → Homework effort. The chain mediating effect of EF and positive homework emotions was significant (the mediating effect value was 0.05, accounting for 6.10% of the total mediating effect). Therefore, hypotheses 2, 3 and 4 were verified.

**Table 3:** Bootstrap analysis of significance test of mediating effect (*N* = 1776).

Effect pathway	Effect size	BootSE	Bootstrap 95% confidence interval		Percentage
			BootLLCI	BootULCI	
Total effect	0.82	0.09	0.64	0.99	100%
Direct effect	0.34	0.08	0.18	0.50	41.46%
Total indirect effect	0.48	0.06	0.37	0.60	58.54%
Indirect effect 1	0.14	0.03	0.08	0.21	17.07%
Indirect effect 2	0.29	0.04	0.22	0.37	35.37%
Indirect effect 3	0.05	0.01	0.03	0.07	6.10%

BootSE, Bootstrap standard error.

**Figure 4:**
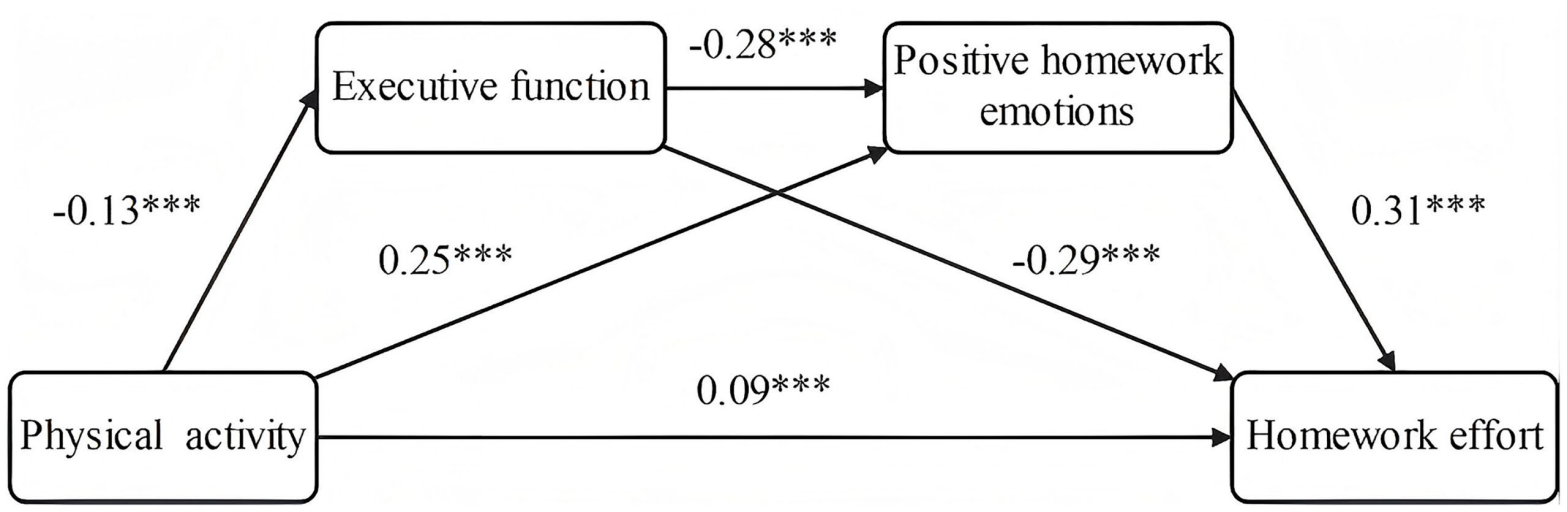
Chain mediation model of executive function and positive homework emotions. ***p < 0.001.

Based on the above analyses, the chain mediation model of physical activity → executive function →positive homework emotions → homework effort is constructed and shown in Figure 4.

## 5 Discussion

This study investigated the relationships among PA, homework effort, EF, and positive homework emotions. It further explored the underlying effect pathways linking these constructs. A review of existing literature indicates that this is the first study to explore the mechanism through which middle school students’ physical activity affects their homework effort. The findings show that PA is directly associated with homework effort and exerts indirect effects via the mediating roles of EF and positive homework emotions. The results offer a theoretical basis and feasible strategies for improving middle school students’ physical health and reducing their academic stress.

The results of the study confirm Hypothesis 1 (H1). In this study, the direct effect size of PA on homework effort among middle school students was relatively small. Previous research indicates that large sample sizes increase the sensitivity of statistical tests, and thus often yielding a small effect size. That said, a small yet statistically significant effect sizes actually suggests that the association between the variables truly exists (Shen et al., 2025). In educational practice, students with higher levels of PA tend to exhibit greater initiative and concentration while completing homework. The significant association between PA and homework effort identified here precisely confirms that PA exerts a positive reinforcing effect on students’ homework effort. This suggests that schools can improve students’ PA levels by ensuring adequate time, frequency, and intensity of physical activities, and it also provides important guidance for optimizing school physical education teaching practices. As a natural and fundamental human behavior, physical activity helps maintain an efficient energy balance (Guo, 2016) and promotes mental health (Yang et al., 2024). However, homework occupies a substantial portion of adolescents’ daily study time, and families, schools, and adolescents generally consider homework essential for improving academic performance (Cooper, 2001). Consequently, increasing homework loads have reduced time for extracurricular physical activity, potentially leading to declines in physical fitness. Homework completion often imposes significant pressure on middle school students, stemming from teacher dissatisfaction, parental criticism, and frustration over task difficulty. These negative emotions can undermine homework effort (Briank, 2005). Physical activity serves as a key intervention for these challenges. Research indicates that physical activity can enhance learning efficiency (Kayani et al., 2018) and mitigate negative emotions from academic stress (Liu et al., 2024), thereby reducing the perceived academic pressure (Solberg et al., 2021). Based on Expectancy-Value Theory, a psychological homework model identifies compliance, investment, and concentration as key predictors of homework behavior (Trautwein et al., 2006). Empirical evidence shows that physical activity enhances adolescent concentration, regardless of activity type (Maltais et al., 2016). Electroencephalography (EEG) studies reveal that physical activity stabilizes response selection and reduces distractions (Hillman et al., 2009). Neuroscience research indicates that physical activity induces structural brain changes by activating the frontal lobes, leading to improved attention, cognition, and learning outcomes (Valkenborghs et al., 2019; Zhou and Jin, 2021). Taken together, these studies provide a theoretical and physiological foundation for the finding that physical activity promotes homework effort.

The results of this study confirm Hypothesis 2 (H2). It is important to note that the Executive Function Scale uses a reverse scoring system, meaning higher scores indicate less favorable executive function. The results show that PA is positively associated with EF, which in turn correlates with greater homework effort. PA influences EF through multiple mechanisms. First, it improves EF by enhancing concentration, memory, inhibitory control, working memory, and cognitive flexibility (Zhong et al., 2022). This matters for academic contexts, as effective learning depends on the coordinated operation of core EF components: working memory, inhibitory control, and cognitive flexibility (Li and Bai, 2008). Empirical studies show that stronger executive function is linked to higher academic performance (Willoughby et al., 2019), and that strong inhibitory control promotes autonomous and efficient homework completion (Liew et al., 2010; Wang et al., 2018). These findings align with our results, validating executive function as a key mediator between physical activity and homework effort. Second, physical activity enhances executive function by stimulating sensory systems (e.g., visual, auditory, motor), improving sensory information processing efficiency. This enhanced processing boosts reaction speed and decision-making abilities (Peng et al., 2018). Over time, this repeated activation and training strengthens sensory system function and improves inhibitory control and working memory (Vazou et al., 2020). Third, since executive function is governed by the prefrontal cortex (Marie, 2017), physical activity promotes neuroplasticity in executive control networks (Wang et al., 2023) and enhances prefrontal cortex function (Su et al., 2023; Wang et al., 2023; Zhong et al., 2022). Together, these mechanisms provide a theoretical basis for the mediating effect of executive function between physical activity and homework effort.

The results of this study confirm Hypothesis 3 (H3). The study found that the pathway linking PA to homework effort via positive homework emotions accounts for the largest proportion of the total effect size. Previous studies have established a significant correlation between physical activity and positive emotions (Pasco et al., 2011) and have shown that regular participation in physical activity can foster positive emotions (Long et al., 2021). Homework emotions refer to the most immediate emotional experiences that students generate in the context of completing homework tasks (Thomas et al., 2012). Existing studies have confirmed that homework emotions constitute a crucial individual factor influencing students’ homework effort (Liu et al., 2013). Specifically, positive homework emotions can positively predict the level of homework effort; students reporting higher levels of pleasant emotional experiences tend to invest more effort in their homework (Ulrich and Oliver, 2008; Liu et al., 2016). These findings are consistent with the results of this study. Neurophysiology research provides a biological basis for how physical activity affects emotions. Studies suggest PA induces pleasure by activating the opioid system in prefrontal-limb brain regions – areas crucial for emotional regulation (Boecker et al., 2008). It also promotes dopamine release and reduces adrenaline secretion, thereby alleviating negative emotions and fostering positive ones (Si et al., 2025). These mechanisms explain the relationship between physical activity and positive homework emotions. Therefore, this finding is physiologically supported and aligns with the understanding that positive emotions predict greater homework effort. Thus, positive homework emotions emerge as the strongest mediating factor in the model of this study. PA can quickly mitigate students’ negative emotions and directly evoke pleasure and initiative during homework completion. Such immediate emotional arousal exerts a more direct effect on increasing students’ homework effort.

The results of this study confirm Hypothesis 4 (H4). The independent mediating roles of executive function and positive homework emotions are supported by theoretical and physiological evidence. Empirical research clarifies the relationship between these two mediating variables. Simply put, EF is associated with academic emotions: stronger EF correlates positively with positive emotions and negatively with negative emotions (Xing et al., 2023), which aligns with the results of this study. These findings align with the results of this study. The physiological mechanism involves interactions between the prefrontal cortex and anterior cingulate cortex, which regulate sub-cortical emotion-generating systems to modulate emotional responses (Sun et al., 2020). Research further shows that physical activity shapes emotional valence by sustaining optimal activation in the left frontal lobe (associated with positive emotions) and inhibiting the right frontal lobe (linked to negative emotions) (Long et al., 2021). This neural and physiological evidence aligns with our finding of a positive correlation between executive function and positive academic emotions. Moreover, these physiological mechanisms provide a basis for the chain-mediating role of EF and positive homework emotions, as verified in the present study.

In addition, previous research has constructed a two-dimensional model of homework habit based on the characteristics of Chinese education system: academic procrastination and academic diligence (Yin and Wu, 2021). This model delineates a dual influence of the local educational context on students’ homework habits: first, a tendency to delay tasks related to homework; second, a capacity for proactive engagement in these tasks.

This study has certain limitations: (1) the sample data were collected from provincial capitals in China, without considering the impacts of factors such as family background, economic status, and regional differences. (2) PA levels were assessed using only self-reported questionnaires, which is a limited measurement approach. Future studies could incorporate instruments and equipment to monitor exercise duration, intensity, and load more scientifically, thereby accurately evaluating students’ physical activity levels. While the data collected via the PAQ may be subject to certain biases, the scale assesses overall PA levels as a continuous variable through a scoring method, which greatly enhances the operational feasibility of the present study. Moreover, all items of the PAQ are designed to reflect the authentic profile of individuals’ physical activity to the greatest extent, and its reliability and validity have been fully verified (Guo, 2016).

The findings of this study suggest that schools, teachers, and parents should attach great importance to students’ level of physical activity. Maintaining a relatively high level of physical activity not only promotes the development of students’ executive functions, but also effectively alleviates student’s academic stress and homework-related anxiety via the pleasurable experiences derived from exercise. When students are in a positive cognitive and emotional state, their motivation to invest time and energy in completing their homework increases significantly. Therefore, schools need to guarantee students’ physical activity time on campus and ensure their access to sports facilities. Physical education teachers should design scientifically rigorous activity with appropriate exercise frequency and intensity. Finally, parents must ensure that students maintain consistent and regular physical activity routines outside of school hours.

## 6 Conclusion

A positive correlation exists among the four variables: PA, EF, positive homework emotions, and homework effort. PA, EF, and positive homework emotions are all potential positive predictors of homework effort. PA is a potential direct predictor of homework effort; it can also exert an indirect effect on homework effort through the mediating roles of EF and positive homework emotions. Specifically, three mediating pathways were identified: the EF-mediated pathway, the positive homework emotion-mediated pathway, and the chain mediation pathway involving EF and positive homework emotions. Among these, the effect size of PA on homework effort via positive homework emotions was the largest. The chain mediation model of EF and positive homework emotions in the relationship between PA and homework effort reveals a novel mechanism underlying the association between PA and homework effort among middle school students, which provides practical implications for enhancing adolescents’ homework effort and academic performance. This suggests that future efforts could focus on promoting PA, enhancing EF, and fostering positive homework emotions to improve adolescents’ homework effort.

## Data Availability

The raw data supporting the conclusions of this article will be made available by the authors, without undue reservation.
